# Estimating the total mortality of seabirds following a marine heat wave

**DOI:** 10.1111/cobi.70273

**Published:** 2026-04-22

**Authors:** Jennifer L. Lavers, William Fulton, Silke Stuckenbrock, Alexander L. Bond

**Affiliations:** ^1^ Adrift Lab, Underwood, Lutruwita/Tasmania Australia; ^2^ Bird Group The Natural History Museum Tring Hertfordshire United Kingdom of Great Britain and Northern Ireland; ^3^ Gulbali Institute Charles Sturt University Wagga Wagga New South Wales Australia; ^4^ Centre for Marine Studies Living Ocean Palm Beach New South Wales Australia

**Keywords:** beached birds, citizen science, community science, mass mortality, muttonbirds, ocean warming, wreck, aves varadas, calentamiento oceánico, ciencia ciudadana, comunidad científica, mortalidad masiva, naufragio, pardela, 搁浅鸟类, 公民科学, 社区科学, 大规模死亡, 短尾鹱, 海洋变暖, 海鸟尸骸

## Abstract

Marine heat waves detrimentally affect a range of marine species, including seabirds, and are increasing in frequency and severity. When thousands of dead seabirds wash up on beaches, the public becomes concerned. However, the number of dead birds recorded on beaches is only a fraction of the total mortality; most birds perish at sea. As a result, estimates of total mortality are scarce, and this impedes the ability to determine how such mortality events affect populations. Community science programs can greatly enhance the geographic or temporal scale of studies, which can be critical when mortalities or changes take place over large distances or many months. Using three community science data repositories, we examined the number and composition of seabirds found dead on beaches in eastern Australia during the 2023–2024 marine heat wave. Mortality estimation models developed for other sectors were refined using measures of searcher efficiency and carcass persistence for beach‐washed birds. Total mortality of sable shearwaters (*Ardenna carneipes*) and short‐tailed shearwaters (*Ardenna tenuirostris*) was >13,900 and >608,000, respectively. The loss of these birds, in total more than 629,000 individuals, highlights the increasingly perilous marine environment in which many marine species now exist.

## Abstract (es)

Estimación de la mortalidad total de aves marina después de una onda de calor oceánica

**Resumen:**

Las olas de calor oceánico dañan a diversas especies marinas, incluidas las aves, y su frecuencia y gravedad van en aumento. Cuando miles de aves marinas muertas aparecen en las playas, la población se alarma. Sin embargo, el número de aves muertas registradas en las playas es solo una fracción de la mortalidad total ya que la mayoría de las aves mueren en el mar. Por esto, son pocas las estimaciones de la mortalidad total, lo que dificulta la capacidad de determinar cómo afectan estos episodios de mortalidad a las poblaciones. Los programas de ciencia ciudadana pueden ampliar considerablemente la escala geográfica o temporal de los estudios, lo que es fundamental cuando las muertes o los cambios se producen a lo largo de grandes distancias o durante muchos meses. Utilizamos tres repositorios de datos de ciencia ciudadana para analizar el número y la composición de las aves marinas encontradas muertas en las playas del este de Australia durante la ola de calor oceánico de 2023‐2024. Los modelos de estimación de mortalidad desarrollados para otros sectores se perfeccionaron con medidas de la eficiencia de los buscadores y la persistencia de los cadáveres de las aves arrastradas a la playa. La mortalidad total de las pardelas sombrías (*Ardenna carneipes*) y las pardelas de cola corta (*A. tenuirostris*) fue de >13 900 y >608 000, respectivamente. La pérdida de estas aves, en total más de 629 000 individuos, resalta el entorno cada vez más peligroso en el que viven actualmente muchas especies marinas.

## 【摘要】

海洋热浪对包括海鸟在内的许多海洋物种造成了负面影响, 且其发生频率和严重程度正在加剧。当数千只死亡海鸟被冲上岸时, 公众会感到担忧。然而, 在海滩上记录到的死亡鸟类数量仅占总死亡量的一小部分;大多数鸟类是在海上死亡的。因此, 关于鸟类死亡总数的估计数据十分匮乏, 阻碍了研究者确定此类死亡事件对种群的影响。公民科学项目可以极大地扩展研究的地理和时间尺度, 当死亡事件或种群变化发生在广阔的地理范围或长达数月的时间内时, 公民科学的贡献至关重要。我们利用三个公民科学数据集, 分析了2023‐2024年澳大利亚东部发生海洋热浪期间, 海滩上发现的死亡海鸟数量及其组成。我们结合搜寻效率指标和海滩冲上岸鸟类尸体的残留时间数据, 对其他领域开发的死亡率估计模型进行了优化调整。研究发现, 淡足鹱(*Ardenna carneipes*)和短尾鹱(*A. tenuirostris*)的死亡总量分别超过13,900只和608,000只。这些鸟类的死亡总量超过了629,000只, 强调了当前许多海洋物种所处的海洋环境正变得日益危险。**【翻译:胡怡思;审校:聂永刚】**

## INTRODUCTION

The warming of the oceans in the past century due to climate breakdown is likely irreversible (Merz et al., 2023; Ripple et al., 2025). Warming rates are predicted to increase sharply over the next few decades (Cheng et al., 2022; Oliver et al., 2019) and will threaten all areas regardless of protection status (Bruno et al., 2018; Parks et al., 2023). Ocean warming has extensive impacts that pose risks to marine ecosystems and society (Cheng et al., 2022), including an increased risk of marine heat waves (MHWs) (i.e., discrete periods of prolonged anomalously warm water that change marine environment through the vertical movement of heat in the water column [Hobday et al., 2016; Holbrook et al., 2020; Smith et al., 2025]). Globally, the annual number of MHW days has risen by 54% over the past century, and eight of the 10 most extreme MHWs ever recorded occurred after 2010 (Oliver et al., 2019; Smith et al., 2021). The longest MHWs persist for months and have dramatic effects on marine life (Frölicher et al., 2018). Individual events cause large‐scale loss and damage to dozens of species over thousands of kilometers of coastline (e.g., Bennett‐Smith et al., 2025; Edgar et al., 2023; Garrabou et al., 2022; Jones, Parrish, Lindsey, et al., 2024) and significant redistribution of prey (e.g., vertical migration of fishes to cooler, deeper waters; Guibourd de Luzinais et al., 2024). Worryingly, habitat loss due to MHWs likely exceeds projected losses by the year 2100 due to long‐term warming linked to climate change (Welch et al., 2023). Despite this, MHWs remain understudied compared with the gradual effects of ocean warming (Alter et al., 2024; Sampaio et al., 2021).

Monitoring the response of the marine environment to pulse events, such as MHWs, is best achieved through long‐term programs that establish baseline conditions, including background rates of species mortality (Giménez et al., 2024). For example, standardized strandings data collected through Brazil's Monitoramento de Praias da Baciade Santos project have provided comprehensive baseline mortality data for more than 75 marine species and have identified fisheries bycatch as the likely source of sea turtle strandings in this region (Prado et al., 2022). However, many jurisdictions lack such dedicated monitoring programs. In their place, community‐based programs can provide much‐needed data for many coastal species and locations. Using beached bird data from four long‐term citizen science programs, Jones, Parrish, MacCready, et al. (2024) estimated ∼400,000 Cassin's auklets (*Ptychoramphus aleuticus*) perished during the 2014–2016 MHW in the northeast Pacific (Jones et al., 2018). Their mortality estimate for the MHW accounted for the background deposition of auklet carcasses based on the longer‐term dataset (Jones, Parrish, MacCready, et al., 2024).

Numerous factors influence the number of animals deposited on beaches, and a large and often variable proportion of animals that die during at‐sea mortality events may never wash up on a beach (Ford et al., 2014; Piatt et al., 1990). Experimental trials examining the drift patterns of seabird carcasses and similarly sized surrogate drifters (500‐mL bottles wrapped in neoprene) showed that, on average, carcasses remained afloat for an average of 8.2 days (Wiese, 2003) and 17–65% of birds and drifters were later detected on a beach (Ford & Varela, 2011; Martin et al., 2020). All experimental birds and drifters released >40 km from the shoreline were washed out to sea, depredated, or became waterlogged and sank before reaching shore (Ford & Varela, 2011; Martin et al., 2020). For example, during a mass mortality event in the Gulf of Alaska, ∼3500 emaciated common guillemots (*Uria aalge*) were recorded washed ashore with the total mortality thought to exceed 121,000 birds (Van Pelt & Piatt, 1995).

Once on the beach, carcass persistence can vary widely across beaches and seasons depending on factors such as beach aspect, substrate, near‐shore currents, wave intensity, and tidal range (Fowler & Flint, 1997; Van Pelt & Piatt, 1995). Carcasses may also be temporarily returned to the ocean and then be translocated along the beach or returned to the sea permanently (Ford et al., 2006). However, in most cases, bird carcasses are pushed high enough on the beach that they remain in the same position even on an ascending tidal cycle (Byrd et al., 2009; Ford et al., 2006; Van Pelt & Piatt, 1995). On many beaches, scavenging by predators is the primary cause of removal of fresh carcasses (Van Pelt & Piatt, 1995; Zimmerman et al., 2020). Foxes sometimes remove 80–90% of bird carcasses within 48 h of deposition (Byrd et al., 2009; Zimmerman et al., 2020).

The detection of bird carcasses in terrestrial habitats is well studied as part of investigations into avian collisions with human‐made structures, such as wind turbines (Kitano et al., 2023; Smallwood et al., 2018). Less research has focused on the marine context (but see Zimmerman et al. [2020], Fowler & Flint [1997], and Byrd et al. [2009]). In general, detection probability increases with carcass size and plumage coloration and can vary among habitat types (Fowler & Flint, 1997; Zimmerman et al., 2020). On sandy beaches, large, brightly colored birds have the highest rates of detection, and burial of carcasses during storms is a significant factor influencing detectability (Van Pelt & Piatt, 1995).

The total estimated mortality of seabirds from an offshore event can be estimated based on the combination of the direct count of carcasses on beaches, searcher efficiency in locating carcasses, persistence of carcasses on the beach, proportion of coastline searched, probability that mortality at sea results in a carcass making it to land, and probability that a carcass is reported to a central repository. These factors are broadly analogous to other situations in which monitoring of dead individuals is used to extrapolate across levels of uncertainty (e.g., offshore oil spills; Haney et al., 2014) and is well established for terrestrial wind farms (e.g., Kitano et al., 2023).

**FIGURE 1:**
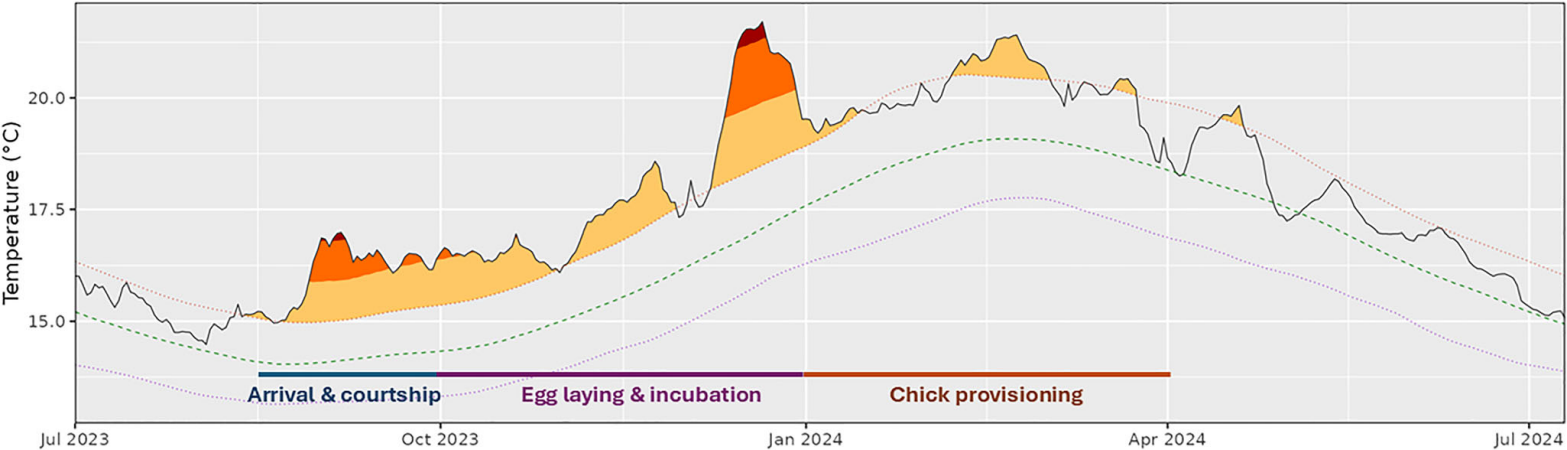
Development of the 2023–2024 marine heat wave off southeastern Australia in the Tasman Sea and Bass Strait (central point −40.125°S, 153.875°E) from 1 July 2023 to 1 July 2024 (yellow, marine heat wave classified as moderate; orange, strong heatwave; red, severe heatwave [Marine Heatwave Tracker, http://whalemap.ocean.dal.ca/MHW/]) and approximate breeding phenology for *Ardenna carneipes* and *Ardenna tenuirostris*.

**FIGURE 2:**
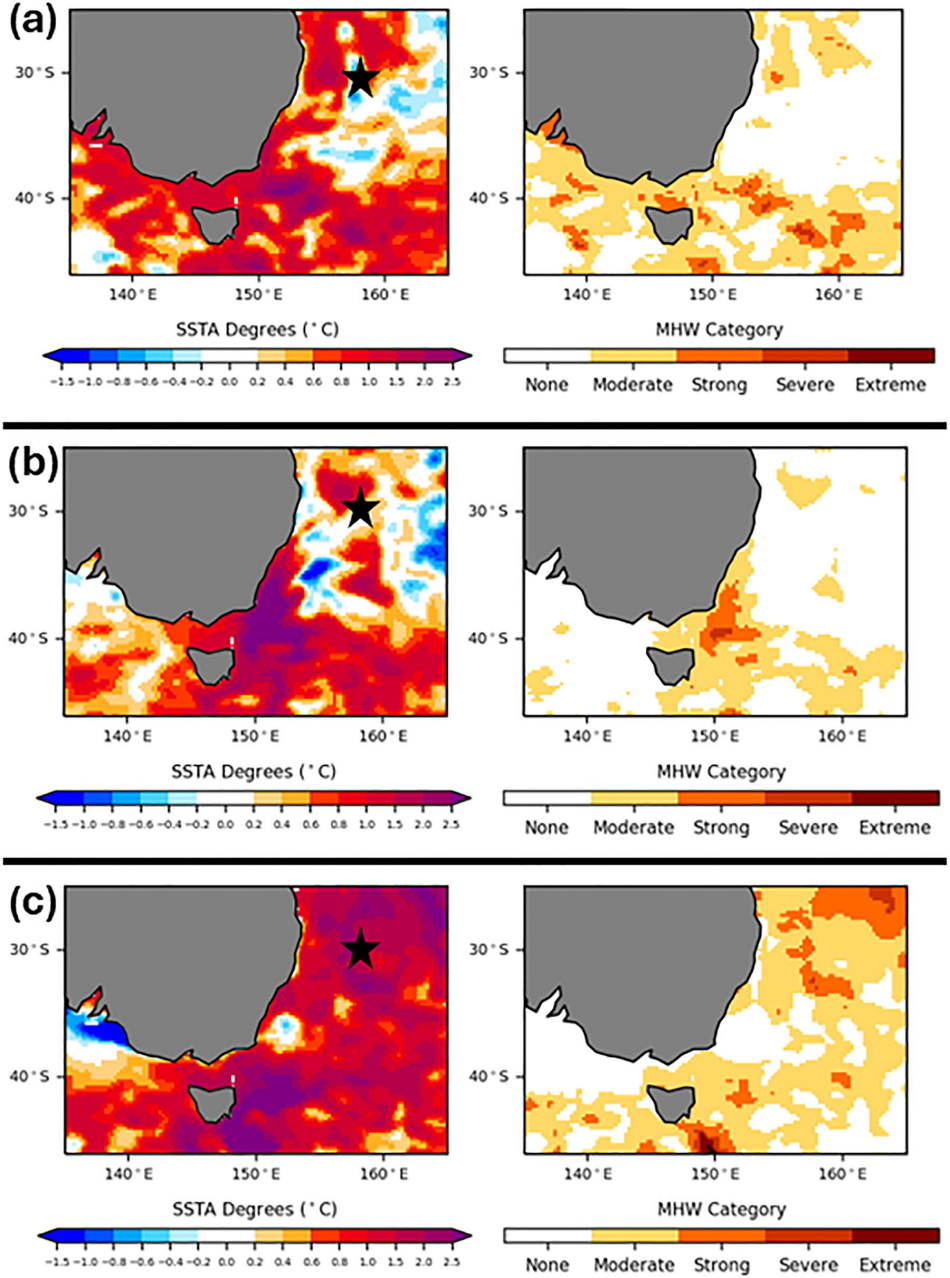
Sea surface temperature anomaly (SSTA) (°C) and marine heat wave (MHW) category for southeastern Australia (star, approximate location of Lord Howe Island, the primary sable shearwater breeding site): (a) shearwater postmigration arrival and courtship period, (b) shearwater egg laying and early incubation, and (c) shearwater chick provisioning. Images and data provided by the Australian Bureau of Meteorology.

In Australia, there are few data for beach‐washed birds and no estimates of total mortality due to major climactic events, such as MHWs. Thus, the community science program rapidly deployed in response to the 2023–2024 MHW off the east coast of Australia (Figures 1 & 2) presents a unique opportunity to investigate the composition and abundance of beached birds and to estimate the total seabird mortality from the MHW. We focused on beach‐washed records for sable shearwater (*Ardenna carneipes*) and short‐tailed shearwater (*Ardenna tenuirostris*), which breed in southern Australia, and used these data to estimate total mortality due to the 2023–2024 MHW. Because migratory species are under increasing pressure and are in decline (BirdLife International, 2024a, 2024b, UNEP‐WCMC, 2026), identifying the conservation implications of two poorly described phenomena (mass mortality of wildlife and MHWs) could not be more timely.

## METHODS

### MHW description

Beginning in late August 2023, forecasts from the Australian Bureau of Meteorology predicted waters off the Tasman Sea, particularly around Tasmania and Victoria, would experience an intense MHW, with the ocean temperature being at least 2.5°C above average from September 2023 to February 2024 (Figures 1 & 2). The MHW peaked in September 2023, which overlapped with the arrival of migrating shearwaters to Australia (BirdLife Australia, 2023a, 2023b), and in December 2023, shortly before the eggs were due to hatch (Figure 1). The sea surface temperature anomaly (SSTA) during this period ranged from 0.6 to 1.0°C but reached extreme levels (>2.0°C) during January 2024 when adults of the two focal species were provisioning their chicks (Figure 2). This MHW persisted for ∼8 months and ended in mid‐March 2024 (Figure 1).

### Seabird description and status

Seventeen seabird species were identified to species level and reported to Adrift Lab from beached bird surveys completed in eastern Australia from 3 November 2023 to 11 May 2024 (Appendix S1). We restricted the analyses to 15 October to 15 February because it encompassed the bulk of mortality, and mortality in the austral autumn could represent postfledging mortality from a variety of sources. Most of the deaths in our analyses were of sable shearwaters (Bond & Lavers, 2024) and short‐tailed shearwaters. Both species have all‐dark plumage and are of similar size (adult body mass 533–692 and 508–740 g, respectively) (BirdLife Australia, 2023a, 2023b). We included the category of ′shearwater sp.′ because species‐level determination was not always possible based on photographs.

### Community science beached bird programs in Australia

At the time of the 2023/2024 MHW, a national coordinated beached bird monitoring program did not exist in Australia. Approximately 4 weeks after the MHW began (November 2023), BirdLife Australia launched their Beach‐Washed Birds portal, part of their Birdata website and mobile phone application. However, there was limited opportunity to communicate this new tool to the broader community in time to capture records and surveys of the ongoing MHW. As a result, uptake was low. Images of beach‐washed birds were sometimes uploaded to well‐known community science websites, such as iNaturalist (Mesaglio & Callaghan, 2021) and the Atlas of Living Australia (Belbin et al., 2021). However, at the time of writing, each of these three platforms provided limited information for contributors to include information on the scope or severity of mass mortality events. For example, typically only one image is uploaded for each record. Thus, most records do not capture information on species composition because the single image often does not show diagnostic features for species identification, such as the bird's beak and feet. Identification also relies on input from the community, and there can be disagreements that are challenging to resolve, especially with taxa that are difficult to identify or when decomposition hinders identification (McMullin & Allen, 2022; authors, personal observation). There is also limited opportunity to report other important data. Individuals who submit data were not required to include the number of people included in the survey or distance walked; thus, search effort cannot be calculated. It is rarely possible to identify instances when only a portion of the beach was searched. Although each online portal includes a free‐form text box, because there is no real‐time moderation of these sites, comments provided by observers are often nonquantitative (e.g., “several” or “many” dead birds are commonly noted), and identifying records of dead or beached birds from trip checklists and other observations is difficult across the huge number of records. The ability of iNaturalist participatory science data to capture the scope and severity of waterbird mortality linked with mass mortality events was assessed. Following the incursion of highly pathogenic avian influenza (HPAI), researchers concluded that iNaturalist can be used to identify the species, timing, and location of relatively high mortality in situations where no other information is available (Taylor et al., 2024). However, iNaturalist alone cannot quantify the magnitude or pinpoint the mechanisms of mortality and therefore is not a viable substitute for comprehensive mortality assessments, such as that undertaken via the Adrift Lab submission process.

Despite the limitations, we included beached bird records from iNaturalist and the BirdLife Australia Birdata web portals collected from October 2023 to May 2024 because these are recognized, long‐standing repositories for observations made by the Australian birding and wider communities. No unique records were found in the Atlas of Living Australia that were not captured elsewhere.

In the weeks leading up to the 2023/2024 MHW (i.e., August–September 2023), marine scientists at the Adrift Lab began reaching out to the birding and broader community via social media, community newsletters, and websites to solicit beached bird data submissions from members of the public. A dedicated Adrift Lab website (https://web.archive.org/web/20251108131435/https://adriftlab.org/beached‐bird‐reports/) with information on how to collect and report beached bird data and photos in a consistent manner was created, following recommendations outlined by Glencross et al. (2021b). Volunteers were asked to take a photo of the overall scene (landscape), plus photos of each beach‐washed bird, taking care to include the beak and feet to enable species identification. Volunteers also reported how far they walked and the number of people in their search team, as well as the total number of birds observed on the beach with dead or dying birds noted separately, where relevant. An Adrift Lab team member was tasked with responding to beached bird data submissions from the community, thereby ensuring any issues with data quality were immediately addressed. In some instances, members of the community were asked to return to the same beach within 24 h to acquire additional photos to assist with species identification, or to undertake targeted experimental surveys (described below).

### Mortality estimation

Because of the highly analogous challenges between MHWs and terrestrial wind energy facilities, we used functions from the R package GenEst (Dalthorp et al., 2018; Simonis et al., 2018) to generate estimates of mortality. These included inputs for searcher efficiency at detecting carcasses, carcass persistence in the environment, a schedule of searches for each site, the density‐weighted proportion of areas searched, and details of the carcasses found, and applied a bootstrap procedure to propagate error through various steps of the estimation. All analyses were undertaken with 1000 bootstrap iterations. Adaptations of the GenEst functions for a beached bird context are detailed below. For further technical details of the analytical framework, see Dalthorp et al. (2018) and Simonis et al. (2018). GenEst requires data tables describing searcher efficiency, carcass persistence, and detection probability, which are described in the subsections below.

### Searcher efficiency

Searcher efficiency, or the probability that a contributor will detect and identify a beached bird carcass, was estimated from the field data. GenEst is based on the assumption that all carcasses used in the efficiency trials are known individually by the study designer and unknown to observers, which was not possible in our field context.

Searcher efficiency was defined as: $\mathrm{Pr}\left\{\mathrm{detect}\;\mathrm{carcass}\;i\;\mathrm{on}\;\mathrm{occasion}\;j\right\}=p_{i}k_{i}^{j-1},$where *p_i_* is the probability that carcass *i* is located on the first search after its arrival on the beach and *k* is the proportional change in searcher efficiency with each successive search (i.e., the fraction by which searcher efficiency decreases on subsequent searches). A value of 𝑘 = 0 asserts that carcasses that are missed on the first search after carcass arrival cannot be discovered in a later search, and 𝑘 = 1 means that searcher efficiency remains constant regardless of carcass age and the number of times a carcass has been missed in previous searches. In cases with high detection probability, such as ours, and with most sites having only one search, *k* will have very little influence on the fatality estimate, so we set *k* = 0 (P. Rabie, personal communication, 2024).

Because our searches were done reactively, we did not have a known number of carcasses in our trials; however, two independent observers simultaneously walked the same beach but in opposite directions to limit the influence of one observer on the other (e.g., through human footprint patterns). Experience level varied across observers, with some volunteers having participated in beached bird surveys previously and others having no relevant experience. Each volunteer recorded the number of carcasses they detected on the beach over eight trials. We chose simultaneous surveys rather than repeat surveys because of the high rates of carcass persistence on beaches (e.g., carcass removal by predators [see below]).

We had eight beaches that were searched simultaneously by two searchers, and leveraged the variation between counts from the two searches to estimate searcher efficiency, as follows. We assumed searcher efficiency was uniform across searches and searchers. For each beach, we simulated carcasses by dividing the observed counts (averaged across two searchers) by hypothetical searcher efficiency values from 0.01 to 0.99. Those virtual carcasses were detected by each of two virtual searchers based on a binomial random number generator for 1000 simulation iterations of each beach. We then recorded the absolute difference between the counts from the two virtual searchers and averaged the absolute differences across the eight beaches. This resulted in 1000 replicate mean average count differences for two virtual searchers on eight beaches, for each of 99 searcher efficiency values. Based on this distribution and the observed mean difference in counts between two searchers, it appears that searcher efficiency was >0.9.

To produce an overall estimate of mortality with functions from GenEst, we therefore created an artificial dataset. For each step, we calculated the number of carcasses assumed to be present (determined as the number of carcasses observed in field trials divided by searcher efficiency). We then determined the difference in observed carcasses between paired searchers and used an estimate of searcher efficiency that best matched the observed difference in carcasses observed in field trials (Appendix S2). This showed that the best approximation of searcher efficiency in our case was >0.95.

Fowler and Flint (1997) assessed carcass detection of king eiders (*Somateria spectabilis*) on sandy and rocky beaches in Alaska and found high detection (∼85–90%) on sandy beaches and lower, but more variable, detection probabilities on rocky beaches. Nearly all of the beaches surveyed in our study were sandy with minimal or no vegetation, and the birds were large and contrasting with the substrate (see details above). Based on this and our own approximations, we opted for a conservative approach that assumed a searcher efficiency of 0.90 and created an artificial dataset of 20 carcasses and two searches to input as the searcher efficiency required for the total mortality estimation functions in GenEst. Although the searcher efficiency model in GenEst can accommodate single searches, we modeled two searches because this reflected our original field searches.

### Carcass persistence

For each carcass, we estimated the day on which it was last recorded as present and the first day it was predicted to be absent. This approach assumed that carcass removal times will be exponentially distributed. This, however, did not provide estimates congruent with field observations, where carcasses persisted for much longer than our modeled estimate (see RESULTS). We therefore used observer remarks and observations to plausibly assume that 33% of carcasses persisted at least 90 days with an exponential decrease over that period. This was based on repeat visits to the same beaches by the same observers. We created an artificial carcass persistence dataset that produced a probability of persistence of 0.33 over 90 days in GenEst.

We ran carcass survival models with four distributions—exponential, Weibull, lognormal, and loglogistic. Model fit was assessed using Akaike's information criterion corrected for small sample size (AIC_c_), and when ranked by AIC_c_ value, those with ΔAIC_c_ >2 from the best fitting model were not considered competitive (Burnham & Anderson, 2002). We created multiple carcass survival models even though we had an artificial dataset because we had no a priori assumptions about the optimal probability distribution.

### Search schedule and detection probability

For each site and each day, we assigned a value of 1 if the beach was searched and 0 if it was not. Most beaches were only searched once, but some were searched up to 11 times (see RESULTS). GenEst assumes that a “clearing search” occurs at *t*_0_ at the same time at each site, and that carcass arrival within each search interval is constant. In our case, carcass arrival was temporally variable, and thus, beaches searched only once (190 of 255 sites) will therefore underestimate mortality during the interval between a hypothetical *t*_0_ and the first search date if searches were conducted before or after the main pulse of mortality or may overestimate if searches were conducted during the period of peak mortality.

To account for the first scenario (underestimation of carcass arrival) for each site, we created a dummy search date for *t*_0_ of 17 October 2023, set *t*_1_ to be the day before our first search on each beach (*t*_2_), and set a final overall search date, *t_f_*, as the latest survey date across the study. We then calculated the proportion of carcasses likely to have arrived between *t*_0_ and *t*_1_ (and therefore be unavailable for counting), between *t*_1_ and each beach's last search, *t_t_* (representing all carcasses that were counted), and between *t_t_* and *t_f_* (reflecting carcasses that may have arrived after the last search on a given beach). We assumed each beach had the same temporal distribution of carcass deposition as overall (i.e., we used the total temporal distribution of carcass deposition). For sites that had a single search, we used *t_t_* = *t*_1_ + 1 to avoid zero proportions.

We recorded the generic carcass detection probability produced by GenEst, but this value was estimated to summarize the overall search campaign and was different from the per‐carcass detection probabilities GenEst uses to estimate total mortality. We used a search interval of 46 days and set the duration to the range of dates covered by our data (i.e., 120 days). From this, we generated the median and 90% confidence interval and the interquartile range.

### Density‐weighted proportion

In GenEst, density‐weighted proportion is the proportion of dead birds that arrive in the search area. We used the categorical data for the at‐sea distributions of sable and short‐tailed shearwaters from Reid et al. (2002) and categorized densities in each 1° block around the east coast of Australia on a scale from 1 (present) to 4 (>40% of total observations) based on their distribution maps. Each degree block was assigned a value of 1–10 based on its distance to shore. At the approximate mid‐point of our beach surveys, 1° of longitude is approximately 91 km. We then fit an exponential curve for both shearwaters to describe the relationship between distance from shore (by degree block) and index of abundance: index of abundance = 95.18e^−0.21 × distance^.

When we set the distance to 0.5 (i.e., 45.5 km), which is the plausible distance from which birds that die at sea could reach land (Martin et al., 2020), we got an index of abundance of 85.7, which is 33% of the summed index of abundance of birds at sea as determined from the distribution maps in Reid et al. (2002). Thus, we assumed that 33% of birds occurred in the 45.5 km belt and were therefore available to be beached. This does not, however, consider the changes in spatial distribution of seabird during MHW events (Cushing et al., 2024), especially with breeding birds tending to be more inshore. This is mirrored in anecdotal observations of shearwaters foraging within sight of the coast in 2023–2024 (which differs from their usual foraging ranges of several hundred kilometers [Cleeland et al., 2014; Reid, 2010]) and in inland freshwater systems. We therefore assumed that 85% of birds at sea would be in the 1–45.5 km bin.

When we assumed that those 85% of birds were uniformly distributed spatially from 1 to 45.5 km and that the probability of beaching was an exponential decay function where 100% of birds from the first 1 km are beached and 0% of birds at 45.5 km are beached (defined as *y* = 433.21 × e^−0.0104^ *^x^* ^+ 5.551^), 78.5% of birds that die at sea from this nearshore bin (1–45.5 km) were expected to end up on a beach. Multiplying this by the 85% of birds at sea in the nearshore bin and available to potentially be beached means of the total number of birds at sea, beaching is expected to capture 66.8% of mortality events. This number was multiplied by the proportion of birds between *t*_1_ and *t_t_* (see “Search schedule and detection probability”) to derive the DWP for each site.

### Overall mortality estimation

Models of searcher efficiency and carcass persistence, along with the density‐weighted proportion and search schedule, were combined to estimate overall mortality. Mortality is calculated starting with a binomial probability function: X∼binomial(M,g), where *X* is the number of carcasses counted on beaches and *g* is the detection probability producing an estimate of mortality, *M*. For technical details of incorporating uncertainty into *g* and therefore determining *M*, see Dalthorp et al. (2018) and Simonis et al. (2018). In our model, we split mortality by species and calculated an overall estimate of mortality.

## RESULTS

During 17 October 2023 to 15 February 2024, beach‐washed seabirds were reported by 209 observers from 246 sites spanning most of the east coast of Australia (Table 1; Appendix S2). Beaches were surveyed from 1 to 12 times with 0 to 301 deceased seabirds detected on each beach. Most specimens were fresh and intact and could be identified to species level; however, 202 shearwaters (4% of all carcasses) with all‐dark plumage could not be identified and were grouped as ′shearwater sp.′. The most common identifiable birds were short‐tailed shearwaters (*n* = 4713) and sable shearwaters (*n* = 55). Twenty‐six carcasses representing 14 seabird species were grouped as the ′other′ category (Appendix S3) and excluded from our analyses due to small sample size.

**TABLE 1:** Summary of records (17 October 2023 to 15 February 2024) of dead seabirds on eastern Australian beaches submitted by community scientists to Adrift Lab and the iNaturalist and BirdLife Birdata web portals during a marine heat wave.

Source	Observers^a^	No. sites^b^	Dates	Total birds^c^
Adrift Lab	209	199	3 November 2023 to 13 February 2024	4765
iNaturalist	68	62	22 October 2023 to 13 February 2024	122
BirdLife Birdata	1	1	17 October 2023	10

^a^ Number of individuals who reported data.

^b^ Number of locations where surveys were conducted (surveys completed at each site from one to 23 times).

^c^ Total number of birds reported across all species.

### Searcher efficiency

Eight beached bird detection trials were completed across seven beaches in New South Wales (Tallow, North Narrabeen, Birdie Fisherman's, Stockton, and Blacksmiths Beach) and Tasmania (Clifton Beach) during 23 November to 6 December 2023 (Table 2). In total, at least 468 carcasses were detected across all trials (Table 2). The number of carcasses was highly correlated among observers (*t*_5_ = 50.05, *r* = 0.999, 95% CI 0.992–0.999, *p* < 0.001). Our model for search efficiency was based on artificial data with *k* fixed at 0. It produced an estimate of *p* = 0.90 (90% CI 0.725–0.968).

**TABLE 2:** Results of searcher efficiency (detection) experiments completed at seven Australian beaches in New South Wales (NSW) and Tasmania (TAS). Counts of seabird carcasses on the beach are provided for each of the observers (Obs).

Location	Distance searched (m)	Date	Observer 1^a^	Observer 2^a^
Tallow Beach, NSW	405	23 November 2023	78	81
North Narrabeen Beach, NSW	426	27 November 2023	25	25
Birdie Beach, NSW (northern end)	523	28 November 2023	91	89
Birdie Beach, NSW (southern end)	514	28 November 2023	65	66
Fisherman's Beach, NSW	682	29 November 2023	15	15
Stockton Beach, NSW	1000	5 December 2023	8	8
Blacksmiths Beach, NSW	950	6 December 2023	180	180
Clifton Beach, TAS	750	2 December 2023	6	6
Total			468	470

^a^ Counts of seabird carcasses on the beach by observer.

### Carcass persistence

The exponential model provided the best fit for carcass persistence, though all distributions were competitive (ΔAIC_c_ < 2.3 for all models) (Appendix S4). This produced a median carcass persistence of 56.9 days (90% CI 46.5–69.6), with 85% of carcasses persisting at last 28 days (Appendix S5).

### Density‐weighted proportion

Using the extrapolations for the density of shearwaters at sea and the relationship between the index of abundance and distance measured in degree blocks (index of abundance = 95.18e^−0.21 × distance^), the likely exponential probability of birds from the first half‐degree block beaching (*y* = e^−0.0921^ *^x^*), and the proportion of birds therefore available. We assumed, however, that a greater proportion of birds would be driven closer to shore during MHW events given their weakened state and observations of shearwaters foraging close to beaches and in freshwater systems. We therefore assumed that 66% of birds that perished at sea during this MHW were available to be beached. When multiplied by the proportion of birds found on individual beaches based on search dates and the temporal patterns of carcass distribution, this produced estimates for the density‐weighted proportion of <0.0001–0.6382 across all sites.

### Overall mortality estimate

Combining these models and propagating uncertainty produced an overall estimate of 629,400 seabirds (90% CI 501,362–847,602), with the majority being short‐tailed shearwaters (96%), followed by sable shearwaters (2.1%) and unidentified shearwaters (1%) (Table 3).

**TABLE 3:** Total estimated mortality for three seabird species during the 2023–2024 marine heat wave off the east coast of Australia.

Species	Median mortality (individuals)	90% CI
Short‐tailed shearwater	608,307	480,183–821,039
Unidentified *Ardenna* shearwater	6290	5317–8238
Sable shearwater	13,923	8362–21,514
Total	629,387^a^	501,362–847,602

^a^ Separate estimate rather than a sum of individual species estimates. Derived from functions in GenEst to split mortality among categories.

## DISCUSSION

The beached bird data collected by community scientists during the 2023–2024 MHW provide some of the first, targeted information on MHW‐related mortality for apex marine predators in Australia (Appendices S3 & S6). This is particularly timely given there are thousands of marine mammals and fish currently washing up on South Australian beaches, with their deaths linked to another MHW (Shepherd, 2025, McAfee and Martin, 2025). The results of our analyses suggest the total mortality during 2023 and 2024 exceeded 629,000 seabirds, with around 96% of the mortalities belonging to a single species, the short‐tailed shearwater (Table 3). Birds observed dead or dying on beaches during the MHW accounted for ∼0.8% of the estimated total mortality (Tables 1 & 3).

Our estimate of mortality may be biased slightly high because the carcass persistence time was often shorter than the interval between searches on many beaches. Because GenEst assumes a constant deposition rate between searches at the individual site level (i.e., if a beach is searched on 1, 20, and 30 June, it is assumed that the rate of carcass deposition is constant from 1 to 20 June and constant, although perhaps different between, from 20 to 30 June), estimates resulting from pulse events may result in high estimates (or low estimates if the main pulse of mortality is missed). Future beached bird surveys should consider the carcass persistence rate when determining search schedules or directing volunteer observers.

The application of analytical frameworks developed for other mortality events, primarily wind energy facilities, to beached bird mortality is promising. Such analyses involve analogous challenges around detection, search effort, and dispersal of carcasses (Dasoler et al., 2020; Martins et al., 2023). Further work to develop these models for beached bird mortality estimates should focus on factors influencing carcass persistence and the density‐weighted proportion, or the proportion of mortality events available to be recorded.

Using beached‐bird carcasses to estimate total mortality is an uncertain exercise (Van Pelt & Piatt, 1995). Only a fraction of birds that die at sea ever wash ashore (Baduini et al., 2001; Wiese, 2003), and they tend to persist for periods of time that are shorter than many survey intervals, if any repeated surveys are conducted at all. Birds are also deposited and removed from surveyed beaches at variable rates due to a range of complex factors, including predation and burial (Fowler & Flint, 1997; Van Pelt & Piatt, 1995). Only a handful of studies have estimated total seabird mortality for MHWs based on observations of carcasses on beaches; however, these data indicate that beached birds account for only a fraction (around 1–3%) of the birds that have died, with total mortality being orders of magnitude greater than what is observed on beaches (Baduini et al., 2001; Jones, Parrish, MacCready, et al., 2024; Piatt et al., 2020). Similarly, in our study we estimated that beached birds represented 0.8% of total mortality. These numbers are comparable to those reported for other at‐sea sources of mortality, such as oil spills, with beach‐washed oiled birds accounting for <5% of total estimated mortality (e.g., Haney et al., 2014; Munilla et al., 2011).

Mass mortality events involving seabirds are often explained by several factors, with the most frequently cited causes being oil spills, starvation from lack of prey, and severe weather (Camphuysen et al., 1999). Although oil spills are often considered to be one of the most significant sources of at‐sea mortality for seabirds (Votier et al., 2005), climate‐driven mass mortality events have been recently shown to result in equivalent, or even greater, mortality. For example, beached bird densities following oil spills are typically 100–140 carcasses/km (Bailey & Davenport, 1972; Lang, 2016). In contrast, >4600 seabird carcasses were recorded per kilometer (across ∼700 km of coastline) during a recent MHW in the North Pacific, and although only 62,000 seabirds were recorded during beach surveys, Piatt et al. (2020) estimated around 1 million seabirds died from the 2015–2016 MHW, with the anomalous warm water referred to as the Pacific blob. Large die‐offs, such as this, have also been recorded elsewhere in the world—for example, ∼400,000 Cassin's auklets died in 2015 and 2016 off the west coast of the United States (Jones, Parrish, MacCready, et al., 2024) followed almost immediately by the death of a further ∼8500 alcids (including Cassin's auklets) in 2017 (Jones et al., 2019). In both cases, the birds’ deaths were attributed to extreme Pacific Ocean temperatures. However, negative impacts of MHWs are not restricted to seabirds, with the 2016 MHW in the Tasman Sea linked with significant mortality of wild Pacific oysters (*Crassostrea gigas*) and blacklip abalone (*Haliotis rubra*) (Oliver et al., 2017). On Lord Howe Island, up to 40% of near‐shore corals died during the 2019 MHW (Moriarty et al., 2023), and extirpations of kelp (*Ecklonia radiata*) have been recorded in Western Australia (Wernberg, 2021). In the North Atlantic, lower phytoplankton productivity as a result of another MHW in 2023–2024 altered the wider food web (Smith et al., 2025). The loss of these species, along with changes to the base of the food web, during MHWs is problematic for seabirds as they underpin the ecological health and functioning of seabird habitats, including foraging locations. Unsurprisingly, the amount of prey consumed by many marine predators, including seabirds, declined significantly in response to MHWs in the Pacific (Gomes et al., 2024). In response, a variety of long‐lasting, carryover effects (e.g., altered foraging or breeding success) have been documented in seabirds following exposure to MHWs (Osborne et al., 2020; Piatt et al., 2020; Romano et al., 2020; Schoen et al., 2024).

**FIGURE 3:**
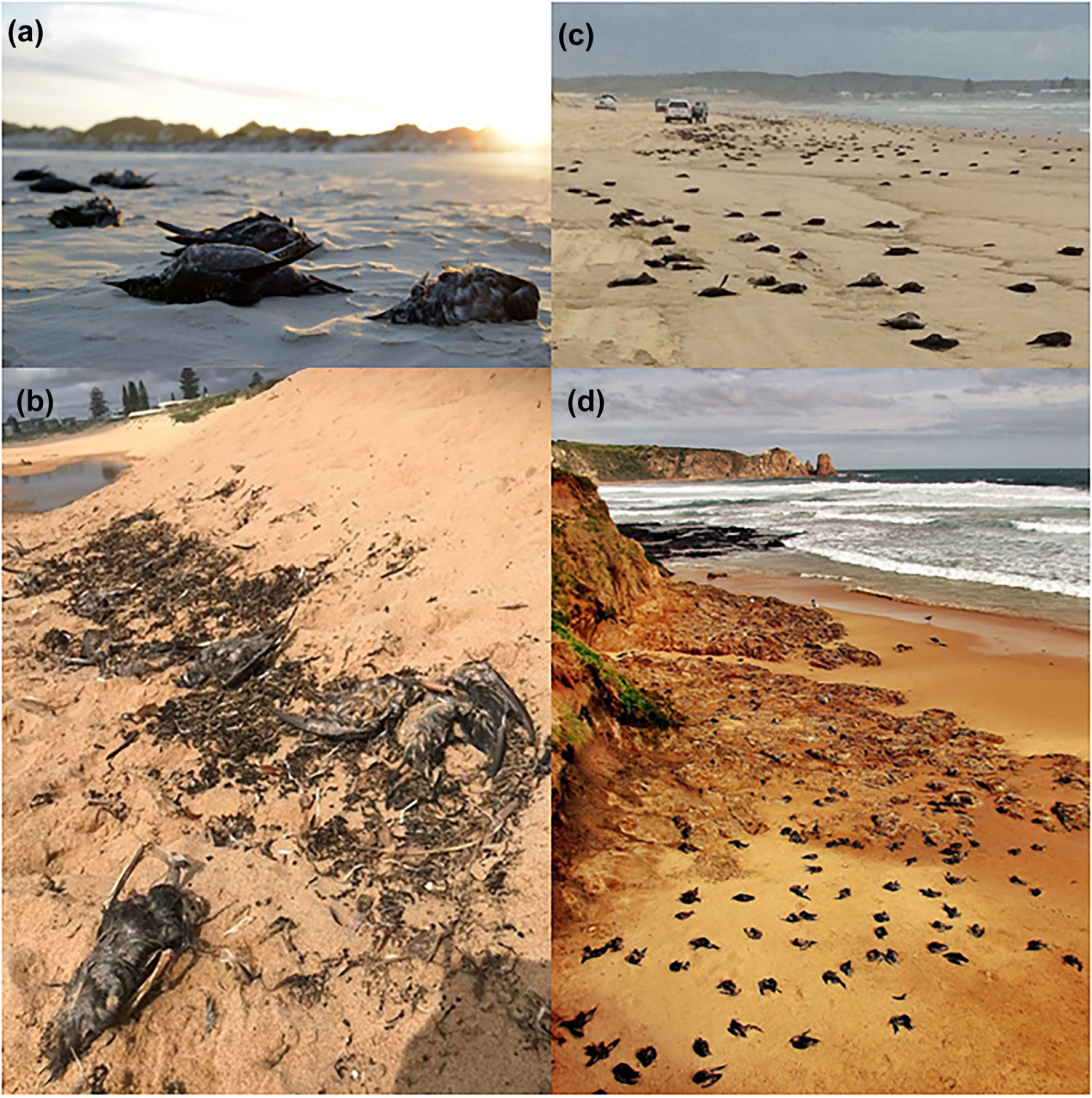
Images of deceased shearwaters on beaches in southeastern Australia: (a) eight shearwaters on One Mile Beach north of Sydney on 11 November 2023 (out of a total of 15 shearwaters reported on the beach that day) (photo by Silke Stuckenbrock), (b) nine dead shearwaters (out of a total of 25) on North Narrabeen Beach near Sydney on 27 November 2023 (photo by Manu Fuchs), (c) hundreds of dead seabirds (almost all short‐tailed shearwaters) on Newcastle Beach (photo by Tracey Austwick), and (d) Cape Woolamai on Phillip Island (panel D) during the 2013–2014 marine heat wave (photo by Anne Morley).

The seabird mortality observed in eastern Australia in 2023 and 2024 is confronting (Figure 3) and of concern for short‐tailed shearwater and sable shearwater population stability; >592,000 and >13,000 birds are estimated to have died during this event, respectively (Table 3). Most of the sable shearwaters would have originated from a single breeding site, Lord Howe Island, which is located approximately 600 km offshore of eastern Australia. Tracking data indicate birds do not overlap in their foraging range: sable shearwaters from Lord Howe forage almost exclusively in the Tasman Sea (where the 2023 and 2024 MHW occurred) (Reid et al., 2012), whereas birds from New Zealand forage farther east in the Pacific (Rayner et al., 2011), and Western Australian birds occupy the Southern and Indian Oceans (Lavers, Lisovski, et al., 2019). Therefore, sable shearwaters breeding in New Zealand and in southwestern Australia are unlikely to have been affected by the MHW, which was confined to Australia's east coast (Figure 2). One of the sable shearwaters recorded beach‐washed during the 2023–2024 MHW was banded on Lord Howe Island (Australian Bird and Bat Banding Scheme data), reinforcing the idea that this site is the source of beached birds. Lord Howe is home to ∼22,000 sable shearwater breeding pairs (Lavers, Hutton, et al., 2019); therefore, mortality of this species in the 2023–2024 MHW accounted for around 29% of the breeding population on this island, though many were likely immature birds that do not return to land until at least 5 years old (Lavers & Bond, 2025). Sable shearwater is listed as near threatened (BirdLife International, 2024b), and the Lord Howe population is in decline (Lavers, Hutton, et al., 2019), so the loss of so many birds in a single event is worrying, especially given the other pressures this species faces, such as chemical and plastics pollution (Lavers et al., 2014, 2021) and bycatch in commercial fisheries (Reid et al., 2012).

Although short‐tailed shearwater populations are estimated at ∼12 million individuals (Silva et al., 2025), many of their populations are poorly monitored or in decline (BirdLife International, 2024b). The loss of ∼592,000 birds during the 2023–2024 MHW (Table 3) is a huge number, but it pales in comparison with the mortality they experienced during another MHW only 10 years ago. During the Austral summer of 2013 and 2014, an estimated ∼200,000 short‐tailed shearwaters washed up on beaches in eastern Australia (Peter & Dooley, 2014; Figure 3, panel c). If one assumes the detection and persistence values in 2013 and 2014 and 2023 and 2024 were similar and that the ∼200,000 short‐tailed shearwaters detected on the beach accounted for only around 2% of the total mortality, then the potential total loss of 10 million shearwaters in 2013 and 2014 would make this the single largest mass mortality event recorded for any seabird and would account for roughly 50% of the total population of this species (Skira et al., 1996). Regardless, two significant mortality events in only a decade clearly indicate that short‐tailed shearwaters are vulnerable to ocean warming and that far more attention should be paid to this species. This is especially true given that the response of many long‐lived seabirds to MHWs is expected to be complex and unpredictable depending on the spatial and temporal scale of heat wave events, as well as their severity (Woehler & Hobday, 2024).

One of the many challenges of working with hyperabundant species, and especially those that are burrow‐nesting and largely nocturnal at breeding sites, such as the short‐tailed shearwater, has been detecting changes when populations are large, widely distributed, and poorly monitored (Major et al., 2013). On many of their breeding islands, populations previously numbered in the millions (Skira et al., 1996). However, with little or no monitoring taking place, suspicion around declines was only highlighted after populations dropped sufficiently low that local communities and Traditional Owners noticed substantially lower numbers of birds compared with previous years. It is worth considering whether, for a hypothetical island with ∼2 million birds, how far numbers would need to decline before one noticed, especially when no monitoring is taking place. Waiting until such a significant decline has taken place has meant researchers and managers have little awareness of what may be driving the decline at a particular site, leaving no choice but to play a dangerous (and avoidable) game of catch up. The total population of short‐tailed shearwaters was estimated at 23 million individuals in 1985 (Skira et al., 1996) at more than 80 sites (BirdLife Australia, 2023b), many of which will have been inadequately surveyed or did not account for occupancy rates, as is the case for other Australian shearwaters (Lavers, 2015; Lavers, Hutton, et al., 2019), so the true population and the conservation status of this iconic and culturally important species are unknown.

**FIGURE 4:**
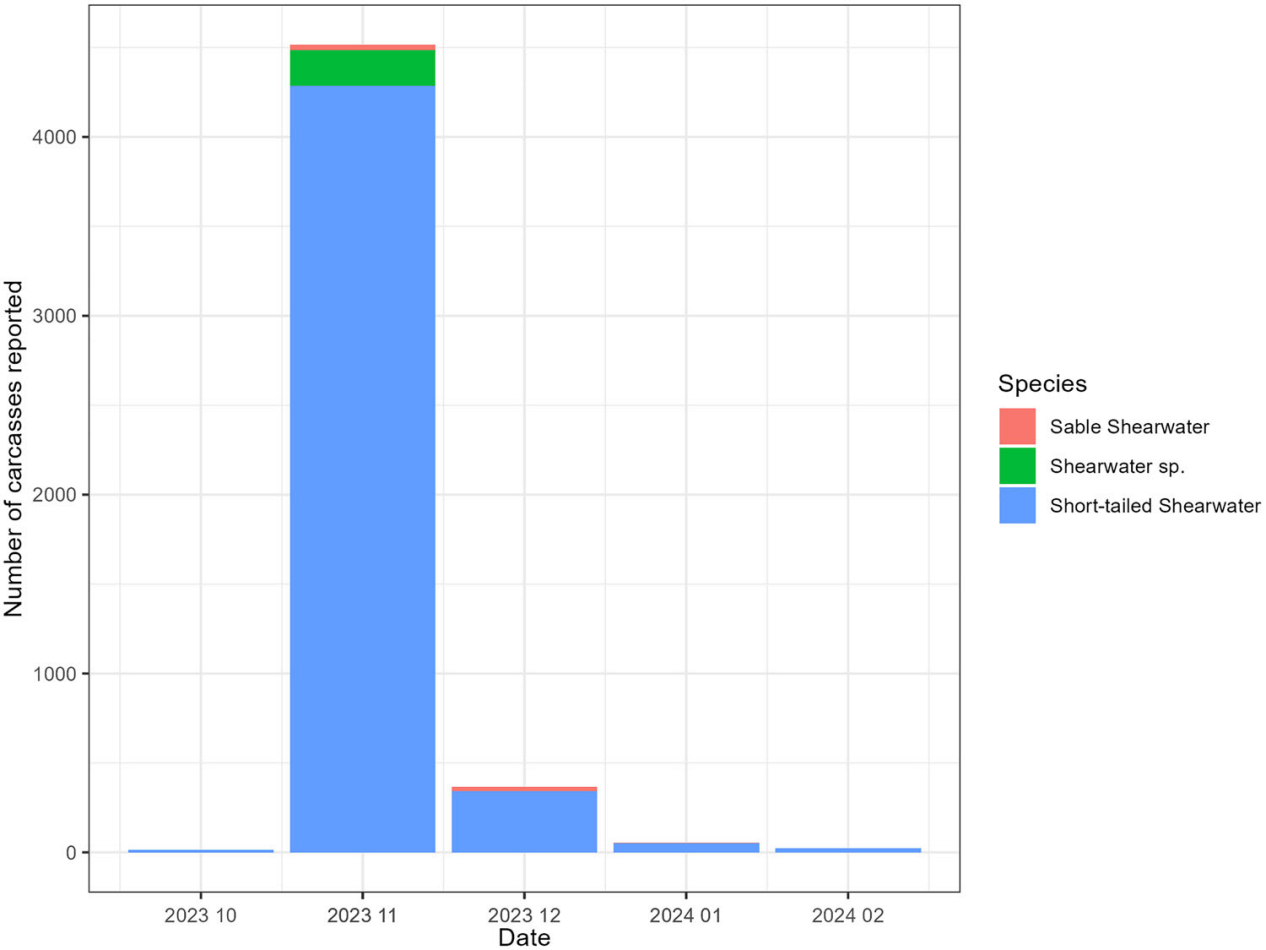
Seabird (mostly shearwaters) carcasses found beach‐washed in eastern Australia from October 2023 to February 2024.

For seabirds, the timing of mortality events can also have a significant influence on population stability. In long‐lived species, populations rely on high survivorship of adult breeding birds and most species have limited capacity to recover from serious perturbations that affect this life stage (Hamer, 2001; Renner et al., 2024). The age class of the two shearwater species affected by both the 2013 and 2014 and the 2023 and 2024 MHWs in Australia were almost entirely adult birds (chicks fledge in April and May) (Figure 4). Thus, the shearwater populations will undoubtedly experience negative consequences, such as disrupted breeding due to loss of one or more members of the pair. Unsurprisingly, short‐tailed shearwater breeding success is significantly lower during MHW years (Glencross et al., 2021a), indicating that carryover impacts affect birds that survive the heat wave.

In Australia, threatened species management is guided by species‐specific conservation advices and recovery plans. However, a review of the “climate readiness” of these recovery plans showed there is a “climate change gap” in both the threat assessment and recommended recovery actions under the *Environment Protection and Biodiversity Conservation* (EPBC) *Act* 1999 (Reynolds et al., 2021). Climate change impacts, including MHWs, were omitted from the conservation advice and recovery plans for 54% of threatened species and ecological communities (*n* = 334) (Reynolds et al., 2021). Furthermore, even in conservation documents that included climate change impacts, the threat analysis tended to be brief and generalized, and the actions recommended to mitigate the impacts were limited (Reynolds et al., 2021). As migratory birds, the two species featured in this study are recognized as “matters of national environmental significance” under Australia's EPBC Act (Department of Environment, 2013). Thus, the disparity between climate change risk and the knowledge and tools required to implement effective recovery plans for threatened species contradicts the premise of Australia's conservation documentation.

Mass mortality events linked with MHWs are increasing in frequency and magnitude in accord with climate change predictions (Frölicher et al., 2018; Oliver et al., 2019), but these are not the only pressures marine animals face. Infectious diseases, such as HPAI (Morin et al., 2018), and other sources of mortality directly linked with a warming climate (e.g., toxic algal blooms; Van Hemert et al., 2020) are also on the rise, and in the absence of robust baseline data, it will become increasingly difficult to disentangle the deaths of animals from the various, often overlapping, causes. At the time of the 2023–2024 MHW, HPAI was not documented in Australia (Wille et al., 2024) and no other anomalous marine events were reported along the east coast. Thus, the seabird mortality we described can be largely, if not entirely, ascribed to the MHW. Although rigorous baseline data do not exist for beach‐washed birds in Australia due to the lack of a dedicated monitoring program, the long‐term citizen science data available through iNaturalist indicated beach‐washed records of the two study species were scant prior to the MHW and that distinct increases in beach carcass density (i.e., wreck events) (Figure 4) are captured at least in part by public reporting.

To adequately respond to emerging and accelerating environmental crises, one must first acknowledge the factors driving observations. In the case of beach‐washed seabirds, a raft of explanations have been proposed, some of which have been substantiated over subsequent years, whereas others are questionable and have little supporting evidence. In recent years, most seabirds caught up in mass mortality events have been emaciated, indicating a lack of prey is a key factor in their deaths (Baduini et al., 2001; Haman et al., 2013; Jones et al., 2019; Piatt et al., 2020). This was also the case for the two shearwater species affected by the 2023–2024 MHW in Australia. Community members and wildlife care groups consistently reported birds were in extremely poor condition (authors, personal observation).

Severe storms and strong winds have been proposed as a possible explanation for the birds’ deaths (reviewed by Camphuysen et al. [1999]), but for most seabird species, this explanation is inconsistent with the evidence. Seabirds, especially shearwaters, thrive in some of the harshest marine environments on our planet, with most species being highly dependent on strong winds in some areas, such as the Southern Ocean, for their migration and daily foraging trips. Indeed, ocean‐going seabirds are so highly adapted for windy environments that they often seek them out (Ventura et al., 2024) and have evolved special adaptations, such as tendons in their shoulder joints, to catapult them vast distances at great speed (Nourani et al., 2023). Shearwaters can survive in the eye of a hurricane for 11 h at an altitude of up to 4700 m and winds exceeding 200 km/h (Shiomi, 2023; Ventura et al., 2024). Although some studies indicate wind can negatively affect seabirds, what they typically refer to is low wind conditions that force seabirds to expend additional energy during flight (Furness & Bryant, 1996). Given this information, it is disappointing to see government departments and reputable wildlife organizations citing weather or nature as the cause of seabird deaths in Australia (e.g., DPIE, 2013; WHA, 2023). Strong winds can certainly contribute to an increased number of seabird carcasses being driven onto beaches, but the reason they are emaciated or died is something entirely different. Starvation takes weeks, not hours or days (Kitaysky, 1999), and so the conditions that result in many thousands of seabirds washing up on beaches spanning >2500 km of Australia's coastline have almost nothing to do with localized or short‐term pulses of high winds and everything to do with one of the most significant MHWs Australia has ever experienced (Figures 1 & 2).

Seabirds, and other marine wildlife, face a multitude of cumulative and often synergistic environmental crises, many of which are rapidly accelerating in their frequency and severity (Fletcher et al., 2024; Guibourd de Luzinais et al., 2024; Starko et al., 2025). Marine biomes face, or may have already exceeded, numerous critical tipping points including plastic, chemical, and nutrient pollution (Fletcher et al., 2024; Lavers et al., 2022; Persson et al., 2022; Richardson et al., 2023). The temperate, east coast waters of Australia, where the 2013‐2014 and 2023‐2024 MHWs took place, is a well‐known global hotspot for marine biodiversity, but these same waters are now experiencing well‐above global average rates of ocean warming due to climate change (Wernberg et al., 2011). This background trend of warming waters, and the pressures that arise, is made worse by predictions of more frequent, intense, and longer‐duration MHWs for Australia (Kajtar et al., 2021). Indeed, the magnitude of these events has been intensifying for birds, fishes, and marine invertebrates, and as pressures on populations mount, there comes a concurrent rise in disease emergence and events produced by multiple interacting stressors (Fey et al., 2015). As the proverbial canary in the (marine) coalmine, seabirds have been screaming to be heard for decades (Khan, 2018); now they lie silent on beaches while societies dither on climate policy (Crowley, 2021).

## Supporting information



Supporting information
