# Supplementary material for: Estimating the total mortality of seabirds following a marine heat wave
Source: Conserv Biol. 2026 Apr 22;40(5):e70273. doi: 10.1111/cobi.70273 (PMC13613565; doi:10.1111/cobi.70273)
Supplement: Supplementary file 1 [file COBI-40-e70273-s001.docx]

**Estimating the total mortality of seabirds following a marine heatwave event**

**Supplementary Materials**

**Carcass persistence**

To estimate carcass persistence rates on beaches, we initially summarised data from the nine previously published studies (Table S2) as none were available for Australia. For each study, we used the reported mean daily survival rate of carcasses, the study duration in days, and the overall number of carcasses to reconstruct datasets and estimate the number of carcasses remaining for each day assuming a constant survival rate over time until we reached the end of the study period, or all carcasses were predicted to have been removed. Any residual difference between the total number of carcasses lost and the sample size (e.g., due to rounding errors) was assumed to have survived the duration of the study. When we modelled the persistence of 1137 carcasses using data from the literature, the model with a loglogistic distribution provided the best fit and estimated a median carcass persistence of 1.537 days (90% CI: 1.446-1.713 days), with only 15.5% of carcasses expected to persist over 28 days. This produced results that were contradictory to our limited observations where most carcasses persisted for 28 days or longer (see Results), and so we adopted the approach outlined in the main text.

**APPENDIX S1.** Geographic boundary for the individual sampling locations included within this study. The straight line distance between the most northerly and southerly sites are 2,025 km.

|  | Site name | Latitude | Longitude |
| --- | --- | --- | --- |
| Most northerly site | K’Gari (Fraser Island), Queensland | -25.22 | 153.26 |
| Most southerly site | Dodges Ferry, Tasmania | -42.86 | 14.62 |
| Most easterly site | Cabarita Beach, Queensland | -28.34 | 153.58 |
| Most western site | Deep Creek, Fleurieu Peninsula | -35.65 | 138.25* |

* Only one seabird (a Short-tailed Shearwater) was recorded from South Australia during Oct 2023 to May 2024; no other beached birds were reported from this state.

**APPENDIX S2** Simulated estimates of differences in observer searcher efficiency based on carcass observations in field trials over a range of values for searcher efficiency (SEEF).

**APPENDIX S3.** Summary of beached birds categorised as “other” in our dataset. Observations were submitted by community scientists to Adrift Lab, but were excluded from our estimates of total mortality due to small sample size. No. recorded = the total number of birds reported during the 2023/2024 marine heatwave in eastern Australia.

| Species | No. recorded |
| --- | --- |
| Australasian Gannet *Morus serrator* | 12 |
| Little Penguin *Eudyptula minor* | 5 |
| Wedge-tailed Shearwater *Ardenna pacifica* | 4 |
| Fluttering Shearwater *Puffinus gavia* | 3 |
| Silver Gull *Chroicocephalus novaehollandiae* | 3 |
| Pied Cormorant *Phalacrocorax varius* | 2 |
| Little Black Cormorant *Phalacrocorax sulcirostris* | 2 |
| Black-faced Cormorant *Phalacrocorax fuscescens* | 1 |
| Great Cormorant *Phalacrocorax carbo* | 1 |
| *Phalacrocorax* spp. | 2 |
| Red-tailed Tropicbird *Phaethon rubricauda* | 1 |
| White-headed Petrel *Pterodroma lessonii* | 1 |
| Storm-petrel *spp.* | 1 |
| Albatross *spp.* | 1 |
| Common Diving Petrel *Pelecanoides urinatrix* | 1 |
| Diving petrel *spp.* | 1 |
| Kelp Gull *Larus dominicanus* | 1 |
| Laridae *spp.* | 3 |
| Australian Pelican *Pelecanus conspicillatus* | 1 |

**APPENDIX S4**. Models evaluated for estimates of carcass persistence.

| Model | Location formula | Scale formula | AICc | ΔAICc |
| --- | --- | --- | --- | --- |
| exponential | l ~ constant | NULL | 726.77 | 0 |
| weibull | l ~ constant | s ~ constant | 728.44 | 1.67 |
| loglogistic | l ~ constant | s ~ constant | 728.59 | 1.82 |
| lognormal | l ~ constant | s ~ constant | 729.11 | 2.34 |

**APPENDIX S5**. Estimates of carcass persistence from the GenEst model.

| Time (days) | % carcasses present  (median & 90% CI) |
| --- | --- |
| 1 | 0.994 (0.993-0.995 |
| 3 | 0.982 (0.978-0.985) |
| 7 | 0.959 (0.950-0.966) |
| 14 | 0.919 (0.903-0.933) |
| 28 | 0.847 (0.818-0.873) |

**APPENDIX S6.** Carcass persistence rates from the literature used in this study.

| Study | Location | Species | Mean daily survival rate | Study duration (days) | Number of carcasses | Notes |
| --- | --- | --- | --- | --- | --- | --- |
| (Camphuysen 1989) | Netherlands | Alcids | 0.98 | 120 | 44 |  |
| (Jones 1989) | Alaska | Oiled birds (mostly guillemots) | 0.84 | 2 | 198 |  |
| (ECI 1991) | Alaska | Radio-tagged seabirds | 0.80 | 8 | 9 |  |
| (ECI 1991) | Alaska | Radio-tagged auklets | 0.47 | 7 | 23 |  |
| (Bodkin & Jameson 1991) | California | Seabirds | 0.96 | 330 | 71 |  |
| (Burger in ECI 1991) | British Columbia | Seabirds | 0.54 | 4 | 12 |  |
| (Van Pelt & Piatt 1995) | Alaska | Guillemots | 0.93 | 100 | 398 |  |
| (Fowler & Flint 1997) | Alaska | King Eiders | 0.72-0.99 | 5 | 179 | Weighted assuming equal males and females. Data extracted from Figure 1 |
| Byrd et al. 2009 | Alaska | Seabirds | 0.146 (SE: 0.034) | 12 | 112 |  |
| (Jones et al. 2017) | Washington | Seabirds | 0.935 | 730 | 100 | Duration taken to be 2 years; no sample size given to scaled to 100 individuals |

**Supplemental references**

Bodkin JL, Jameson RJ. 1991. Patterns of seabird and marine mammal carcass deposition along the central California coast, 1980-1986. Canadian Journal of Zoology **69**:1149-1155.

Camphuysen CJ. 1989. Beached bird surveys in the Netherlands, 1915-1988. Seabird mortality in the southern North Sea since the early days of oil pollution. Amsterdam, The Netherlands.

ECI. 1991. Assessment of direct seabird mortality in Prince William Sound and the western Gulf of Alaska resulting from the Exxon Valdez oil spill. Anchorage, Alaska.

Fowler AC, Flint PL. 1997. Persistence rates and detection probabilities of oiled King Eider carcasses on St Paul Island, Alaska. Marine Pollution Bulletin **34**:522-526.

Jones IL. 1989. A beached bird survey of Ushagat Island, Barren Islands, Alaska, April 30-May 2, 1989. Anchorage, Alaska.

Jones T, Parrish JK, Punt AE, Trainer VL, Kudela R, Lang J, Brancato MS, Odell A, Hickey B. 2017. Mass mortality of marine birds in the Northeast Pacific caused by Akashiwo sanguinea. Marine Ecology Progress Series **579**:111-127.

Van Pelt TI, Piatt JF. 1995. Deposition and persistence of beachcast seabird carcasses. Marine Pollution Bulletin **30**:794-802.
